# Modulation Signal Recognition Based on Information Entropy and Ensemble Learning

**DOI:** 10.3390/e20030198

**Published:** 2018-03-16

**Authors:** Zhen Zhang, Yibing Li, Shanshan Jin, Zhaoyue Zhang, Hui Wang, Lin Qi, Ruolin Zhou

**Affiliations:** 1 College of Information and Communication Engineering, Harbin Engineering University, Harbin 150001, China; 2 College of Air Traffic Management, Civil Aviation University of China, Tianjin 300300, China; 3 Department of Electrical and Computer Engineering, Western New England University, Springfield, MA 01119, USA

**Keywords:** entropy feature, feature selection, ensemble learning, radar

## Abstract

In this paper, information entropy and ensemble learning based signal recognition theory and algorithms have been proposed. We have extracted 16 kinds of entropy features out of 9 types of modulated signals. The types of information entropy used are numerous, including Rényi entropy and energy entropy based on S Transform and Generalized S Transform. We have used three feature selection algorithms, including sequence forward selection (SFS), sequence forward floating selection (SFFS) and RELIEF-F to select the optimal feature subset from 16 entropy features. We use five classifiers, including *k*-nearest neighbor (KNN), support vector machine (SVM), Adaboost, Gradient Boosting Decision Tree (GBDT) and eXtreme Gradient Boosting (XGBoost) to classify the original feature set and the feature subsets selected by different feature selection algorithms. The simulation results show that the feature subsets selected by SFS and SFFS algorithms are the best, with a 48% increase in recognition rate over the original feature set when using KNN classifier and a 34% increase when using SVM classifier. For the other three classifiers, the original feature set can achieve the best recognition performance. The XGBoost classifier has the best recognition performance, the overall recognition rate is 97.74% and the recognition rate can reach 82% when the signal to noise ratio (SNR) is −10 dB.

## 1. Introduction

With the continuous development of technology, the density of radar signals has increased and the electromagnetic environment has become more and more complex. A variety of countermeasures have been proposed [1]. Various electronic protection measures, the application of new interference technology and new radar signal modulation modes cause great problems for radar emitter recognition. Therefore, it is very important to study the internal characteristics of the signal emitted by radar emitters.

Early radar signal modulation was simple and the signal quantity was small. In this electromagnetic environment, traditional radar emitter recognition was mostly based on pulse description word (PDW). PDW parameters [2] were extracted quickly through the parameter estimation and signal sorting in mixed signals, which achieved sorting and recognition within the wide range of the signal to noise ratio (SNR). Since the single PDW sequence had limitations in analyzing the modulation characteristics, according to the actual data samples obtained, the time domain 12-dimensional characteristic parameters of the pulse sequence in the were listed in [3].

Considering that time domain features are easily affected by noise and interference, the current research has focused more on the transform domain. The time-frequency characteristics of signals were analyzed by the Wigner-Ville method in [4]. A joint time-frequency analysis (JTF) was used to mine the time-varying information of signals to improve the ability of signal analysis for stationary radar signals [5]. Furthermore, the smooth pseudo-random Wigner distribution was proposed, and this highlighted that high time-frequency energy aggregation and less cross term are the key factors for determining the recognition of the radiation source [6].

In recent years, many studies have constructed a more complete set of information from the perspective of feature combination. To utilize the advantages of the entropy feature in the recognition of radiation sources, the sampling entropy (SampEn) and fuzzy entropy (FzzyEn) were extracted from the radiation source to measure the complexity and uncertainty of the signal [7]. In [8], almost 42 dimensional parameters, including phase offset, power spectral density (PSD), Zero delay and cumulant of complex envelopes, and so on were extracted for identification [9].

At present, feature extraction methods are increasingly used in the field of radiation source recognition to meet the requirements of specific environments and for the application of radiation source recognition [10,11,12,13], including the main ridge slice feature of the fuzzy function [14], random projection compression of high dimensional data features [15], and deep learning [16].

For ensemble learning, Adaboost has been used for recognition of source modulations for multiple-input multiple-output two-way relaying channel (MIMO TWRC) with physical-layer network coding (PLNC), and it achieved good performance at acceptable SNR values [17], but it did not have the robust algorithms for recognition of other communication parameters. The Gradient Boosting Decision Tree (GBDT) was used for High Resolution Range Profile (HRRP) target recognition, which showed that GBDT achieved better recognition results and higher calculation efficiency than the Support Vector Machine (SVM) and Naive Bayes classifier [18]. The weighted-XGBoost was used as the model to classify radar emitters, and it achieved better performance than several existing machine learning algorithms [19]. In order to enhance the probability of communication digital signal recognition, ensemble learning was used in [20], which studied eight types of modulation signals. The limitations were that the SNR interval was 3 dB and classification training and testing were performed at each SNR.

In this paper, we focus on the entropy feature extraction and ensemble learning algorithms. Firstly, we extract 16 kinds of entropy features of nine different kinds of digital signals. Then we use three feature selection algorithms, including the sequence forward selection (SFS) algorithm, sequence forward floating selection (SFFS) algorithm and RELIEF-F algorithm, for selecting the optimal feature subset from 16 entropy features. Finally, we use five classifiers, including the *K*-nearest neighbor (KNN) classifier, (SVM) classifier, Adaboost classifier, GBDT classifier and XGBoost classifier to classify the original feature set and the feature subset selected by different feature selection algorithms. By analyzing the simulation results, we find the optimal feature subset, the best selection algorithm and the best classifier.

The paper is organized as follows. Section 2 briefly introduces entropy feature extraction algorithms, feature selection algorithms and classifiers. Section 3 describes the experimental data, methodology and results, which are analyzed in detail. Lastly, Section 4 gives conclusions and possible future research directions.

## 2. Theories and Methods

**Figure 1:**
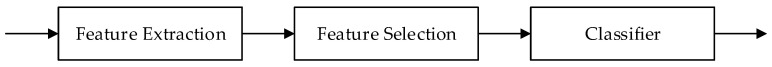
The commonly used recognition framework.

The commonly used recognition framework generally includes three parts, including feature extraction, feature selection and classifier, which is shown in Figure 1. Feature extraction algorithms include entropy features, higher order moment features, the higher order cumulant features, etc. Feature selection algorithms include the SFS algorithm, SFFS algorithm, and the RELIEF algorithm. Classifiers include KNN, SVM, ensemble learning, etc.

### 2.1. Entropy Feature Extraction Algorithm

#### 2.1.1. Common Entropy

Entropy can measure the uncertainty of the value of random variables [21]. There are two general definitions of entropy [22]:

(1) Shannon entropy:

(1) $$H\left(p\right)=H\left(p_{1},p_{2},\ldots ,p_{n}\right)=-\sum_{i=1}^{n}p_{i}\log p_{i}$$

(2) Exponential entropy:

(2) $$H=\sum_{i=1}^{N}p_{i}e^{1-p_{i}}$$

For the signal sequence $X=\left\{x_{1},x_{2},\ldots ,x_{N}\right\}$, the power spectrum entropy [21] is defined as:

(3) $$S\left(\omega \right)=\frac{1}{N}{\left|X\left(\omega \right)\right|}^{2}$$

where, X(ω) is the Fourier transform of the sequence X. Get the probability distribution $p_{i}$, and finally calculate the power spectrum Shannon entropy and power spectrum exponential entropy.

(4) $$p_{i}=\frac{S\left(i\right)}{\sum_{i=1}^{N}S\left(i\right)}$$

The sequence X is segmented to generate matrix A. The singular value spectrum is obtained by the singular value decomposition (SVD). Get the probability distribution $p_{i}$, and finally calculate the singular spectrum Shannon entropy and singular spectrum exponential entropy [21].

(5) $$A=\left[\begin{array}{l} x_{1},x_{2},\ldots ,x_{M}\\ x_{2},x_{3},\ldots ,x_{M+1}\\ x_{N-M},\ldots ,x_{N} \end{array}\right]$$

Wavelet transform is performed on the sequence X to obtain the wavelet coefficients $W_{f}\left(a,b\right)$ of *n* scales. The energy value at the scale i is $m_{i}$, and the probability distribution is $p_{i}$. Finally, the wavelet energy spectrum entropy at the corresponding scale is calculated [21].

(6) $$W_{f}\left(a,b\right)=\frac{1}{\sqrt{\left|a\right|}}\int_{-\infty }^{\infty }x\left(t\right)\psi^{*}\left(\frac{t-b}{a}\right)dt$$

The Fourier transform of the third order cumulant of the sequence X can obtain $B_{x}\left(\omega_{1},\omega_{2}\right)$. $p_{B^{\prime }}\left(\omega_{1},\omega_{2}\right)$ can be obtained after normalization. Finally, the bispectrum entropy [23] is calculated.

(7) $$B_{x}\left(\omega_{1},\omega_{2}\right)=\int_{-\infty }^{+\infty }\int_{-\infty }^{+\infty }C_{3x}\left(\tau_{1},\tau_{2}\right)e^{-j\left(\omega_{1}\tau_{1}+\omega_{2}\tau_{2}\right)}d\tau_{1}d\tau_{2}$$

The approximate entropy [24] is:

(8) $$H_{ApEn}\left(m,r,N\right)=\phi^{m}\left(r\right)-\phi^{m+1}\left(r\right)$$

The sample entropy [25,26] is:

(9) $$H_{SaEn}\left(m,r,N\right)=-\ln\frac{B^{m+1}\left(r\right)}{B^{m}\left(r\right)}$$

The fuzzy entropy [27] is:

(10) $$H_{Fuzzy}\left(m,r,N\right)=\ln\frac{\phi^{m+1}\left(r\right)}{\phi^{m}\left(r\right)}$$

#### 2.1.2. Entropy Based on Time-Frequency Analysis

The Short Time Fourier transform (STFT) [28] is:

(11) $$STFT_{S}\left(t,\omega \right)=\int_{-\infty }^{+\infty }s\left(\tau \right)h^{*}\left(\tau -t\right)e^{-j\omega \tau }d\tau$$

The Smoothed Pseudo Wigner-Ville Distribution (SPWVD) [28] is:

(12) $$SPWVD\left(t,f\right)=\int_{-\infty }^{+\infty }\int_{-\infty }^{+\infty }s\left(t-u+\frac{\tau }{2}\right)s^{*}\left(t-u-\frac{\tau }{2}\right)h\left(\tau \right)g\left(u\right)e^{-j2\pi /\tau }d\tau du$$

The S Transform [29] is:

(13) $$S\left(\tau ,f\right)=\int_{-\infty }^{\infty }x(t)\cdot \frac{\left|f\right|}{\sqrt{2\pi }}\exp\left(-\frac{{\left(t-\tau \right)}^{2}f^{2}}{2}\right)\cdot \exp\left(-j2\pi ft\right)dt$$

The Generalized S Transform [30] is:

(14) $$GST\left(\tau ,f\right)=\int_{-\infty }^{\infty }x\left(t\right)\cdot \frac{\lambda {\left|f\right|}^{p}}{\sqrt{2\pi }}\cdot \exp\left(-\frac{\lambda^{2}{\left(\tau -t\right)}^{2}f^{2p}}{2}\right)\cdot \exp\left(-j2\pi ft\right)dt$$

The Rényi entropy [31] is:

(15) $$R^{\alpha }\left(p\right)=\frac{1}{1-\alpha }\log_{2}\frac{\sum_{i}P_{i}^{\alpha }}{\sum_{i}P_{i}}$$

For the energy entropy [32], first calculate the energy E of the time-frequency submatrix, then calculate the probability distribution $p_{ij}$, and finally obtain the energy entropy.

(16) $$E=\int_{(i-1)\Delta t}^{i\Delta t}\int_{(j-1)\Delta f}^{j\Delta f}s_{ij}\left(f,t\right)dfdt$$

### 2.2. Feature Selection Algorithms

#### 2.2.1. Sequence Forward Selection Algorithm

The sequence forward selection (SFS) algorithm, first proposed by Whitney in 1971 [33,34,35], is also known as the set addition algorithm. It is a bottom up search method. The required feature set needed is first initialized to an empty set. Each time we add one feature to the selected feature set until the required feature set meets the requirement, the feature set obtained is the result of the algorithm running. The statistical correlation between the algorithm features is not fully considered, and it is most likely that the best feature set does not include the feature with the largest contribution (the criterion function value), but only the feature combination with the most common contribution rate. 

#### 2.2.2. Sequence Forward Floating Selection Algorithm

The sequence forward floating selection (SFFS) algorithm [34,36] is a typical bottom up feature selection algorithm based on search strategy, which mainly includes two steps: inclusion and conditional exclusion. Inclusion creates a feature set (an empty set at the beginning), and adds a feature selected from the original feature set according to a specific rule for the created feature set each search. Conditional exclusion selects a feature from the selected feature set and removes the feature from the selected feature set if the feature satisfies the criteria that after the removal of the feature, the classification accuracy based on the selected feature set reaches the maximum and is greater than the pre-removal criteria. The algorithm can avoid the local optimal problem of the feature set to some extent. 

#### 2.2.3. RELIEF-F Algorithm

The RELIEF algorithm, first proposed by Kira in 1992 [37], is a kind of feature weighting algorithm, that is, according to the relevance of each feature and category, the weight of the different features are given, and the features whose weight is less than the threshold will be removed. However, its limitation is that it can only deal with two-class problems, so Kononenko extended it in 1994 [38], and obtained the RELIEF-F algorithm, which can deal with noisy and multi-class data sets.

### 2.3. Classifiers

#### 2.3.1. *K*-Nearest Neighbor Classifier

*K*-nearest neighbor (KNN) was proposed by Cover and Hart [39]. It is an instance-based classification method, which has the advantages of simple principles and wide application [40,41].

The basic principle of the KNN classifier is that, given a sample *x* to be classified and a set of labeled instances, the aim of the classifier is to predict the class label of the sample *x* through the instances. The KNN algorithm calculates the distance between the sample *x* and all samples in the labeled instances by using the distance similarity function, trying to find the *k* targets that are most similar to the sample *x* to be classified, and according to the category of the *k* targets using most votes to decide the class label of sample *x*.

In order to determine the categories of samples, it is necessary to calculate the similarity between samples, and the distance measurement is often used. The distance measurement is used to calculate the distance in the space between the targets after quantization. The larger the distance, the larger the difference between the samples is, i.e., the smaller the similarity. The common distance measurement method is Euclidean distance. 

#### 2.3.2. Support Vector Machine

Support vector machine (SVM), first put forward by Cortes and Vapnik in 1995 [42], is the machine learning system to solve the problem of two-group classification. After many improvements, it has become the mainstream technology for machine learning [43,44].

The basic principle of SVM is to use nonlinear mapping to map input vectors to high-dimensional feature space, and to construct the optimal hyperplane for separation of training data without errors in the high-dimensional feature space.

To map samples to high-dimensional feature space, the choice of kernel function is an important research aspect in SVM classification. If the kernel function is not suitable, it means that the samples are mapped to an unsuitable feature space, which is likely to result in poor performance. The commonly used kernel functions include linear kernel function, polynomial kernel function, Gaussian radial basis function (RBF) kernel function and Sigmoid kernel function [45].

#### 2.3.3. Adaboost

Adaboost is an iterative algorithm first proposed by Freund and Schapire in 1995. Freund and Schapire deduced this new boosting algorithm by using the multiplicative weight-update technique. In boosting algorithms, we do not need to have prior knowledge of the basic weak learning algorithm, and it can adapt to the errors of the weak hypotheses returned by WeakLearn [46,47].

The basic principle of Adaboost is to train different basic classifiers (weak classifiers) with the same training set, and then assemble these weak classifiers to get a stronger final classifier (strong classifier).

Adaboost is a typical boosting algorithm. For this kind of algorithm, we need to consider two questions: the first is how to change the weight or probability distribution of training data in each round; the second is how to combine weak classifiers to create a strong classifier. In response to the first question, Adaboost increases the weight of the samples wrongly classified by the weak classifier in the previous round, and reduces the weight of those samples correctly classified. In response to second question, Adaboost takes a weighted majority vote. Specifically, it increases the weight of the weak classifier with a small classification error rate so as to enable it to play a larger role in the voting and reduces the weight of the weak classifier with a large classification error rate so as that it plays a smaller role in the voting.

#### 2.3.4. Gradient Boosting Decision Tree

The Gradient Boosting Decision Tree (GBDT), first proposed by Friedman, is a type of boosting algorithm, which performs well, has wide application, and can be used to solve classification and regression problems [48,49].

The basic principle of GBDT is that each tree trains the error of the previous tree classification result, that is, the residual of the training result of the previous tree and the true value is the target of the training optimization of the current tree, and the final result of the model is obtained by summing the results of every tree. In GBDT, the weak learner qualifies only for the Classification And Regression Tree (CART) regression tree model. For the fitting of the loss function, the approximate value of the loss is fitted with the negative gradient of the loss function, and then a CART regression tree is fitted.

#### 2.3.5. XGBoost

XGBoost, short for eXtreme Gradient Boosting, was proposed by Tianqi Chen at the University of Washington based on the Gradient Boosting Machine [50]. XGBoost is an extensible machine learning system based on tree boosting designed to be efficient, flexible, and portable. The influence of the system has been widely recognized in many machine learning and data mining challenges.

The biggest feature of XGBoost is that it can automatically use multi-threading for parallel computing while improving the accuracy of the algorithm. XGBoost provides a parallel tree boosting (also known as GBDT), which quickly and accurately solves a lot of data problems. The same code runs on major distributed environment that can solve problems for more than billions of examples. For the traditional GBDT algorithm, only the derivative information of the first order is used. When the current tree is trained, the residual of the previous tree is needed, which is difficult to achieve distributed. XGBoost uses a second order Taylor expansion for the loss function, using both the first and second order derivatives, and for avoiding over-fitting adds the regularization term which can help to smooth the final learnt weights.

## 3. Results and Discussion

### 3.1. Experimental Data

In the simulation experiment, we simulated 9 digital signals including 2ASK, 4ASK, 2FSK, 4FSK, 8FSK, BPSK, QPSK, 16QAM and 32QAM. Signal parameter setting were: carrier frequency $f_{c}=4\;\mathrm{MHz}$, sampling frequency $f_{s}=4\times f_{c}$, MFSK(M = 2, 4, 8) signal initial frequency $f_{1}=1\;\mathrm{MHz}$, frequency deviation Δf=1 MHz. Signal length $N_{s}=2048$, digital signal symbol rate $R_{s}=1000\;\mathrm{Sps}$ (Symbol per second, Sps). The baseband signal is random code, and the number of symbols is 125. The digital signal is formed by rectangle pulse, and the roll-off factor is 0.5. The noise is gaussian white noise.

Data sets include a training set and test set. The training set contains 46,800 samples: the signal to noise ratio (SNR) is from −10 dB to 15 dB, each of which has 200 samples per signal. The test set contains 46,800 samples: the SNR is from −10 to 15 dB, each of which has 200 samples per signal.

### 3.2. Experimental Methodology

We extracted 16 kinds of entropy features of 9 kinds of digital signals, including the power spectrum Shannon entropy, power spectrum exponential entropy, singular spectrum Shannon entropy, singular spectrum exponential entropy, wavelet energy spectrum entropy, bispectrum entropy, approximate entropy, sample entropy, fuzzy entropy, Rényi entropy of STFT, Rényi entropy of SPWVD, Rényi entropy of Wavelet Transform, Rényi entropy of S Transform, Rényi entropy of Generalized S Transform, energy entropy of S Transform, and energy entropy of Generalized S Transform.

We used three feature selection algorithms, including the SFS algorithm, SFFS algorithm and RELIEF-F algorithm, to select the optimal feature subset from 16 entropy features. The SFS algorithm and SFFS algorithm belong to the Wrapper method. RELIEF-F algorithm belongs to the Filter method. The specific parameters of the SFS algorithm are set as follows: nested KNN classifier, and the nearest neighbor number *k* is set to 5, 10, 15 and 20. The specific parameters of the SFFS algorithm are set as follows: nested KNN classifier, and the nearest neighbor number *k* is set to 5, 10, 15 and 20. The specific parameters of the RELIEF-F algorithm is set as follows: the nearest neighbor number *k* is set to 10, the number of iterations *m* is the number of samples in the training set, and the threshold value of the feature weight is 0.00. According to the data set, the size of the original feature set and the feature subset of each algorithm, the running time of each algorithm, and the classification accuracy of five classifiers on each feature set are recorded. 

We use five classifiers, including KNN, SVM, Adaboost, GBDT and XGBoost to classify the original feature set and the feature subset selected by different feature selection algorithms. The specific parameter of the KNN classifier is set as follows: the nearest neighbor number *k* is set to 7, 12, 5. The specific parameter of the SVM classifier is set as follows: the kernel function is RBF kernel function. The specific parameters of the Adaboost classifier are set as follows: the depth is 12, 12, 11, the learning rate is 0.1, and the number of iterations is 10. The specific parameters of the GBDT classifier are set as follows: the depth is 9, 9, 9, the learning rate is 0.1, and the number of iterations is 10. The specific parameters of the XGBoost classifier are set as follows: the depth is 12, 15, 16, the learning rate is 0.1, and the number of iterations is 10. According to the data set, the simulation time and recognition rate of different classifiers are calculated.

### 3.3. Experimental Results and Discussion

**Figure 2:**
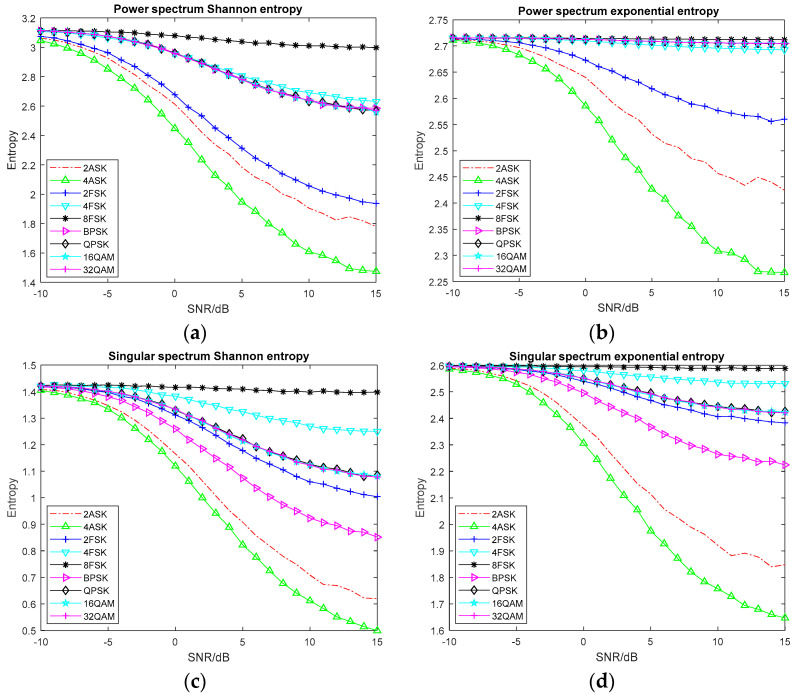

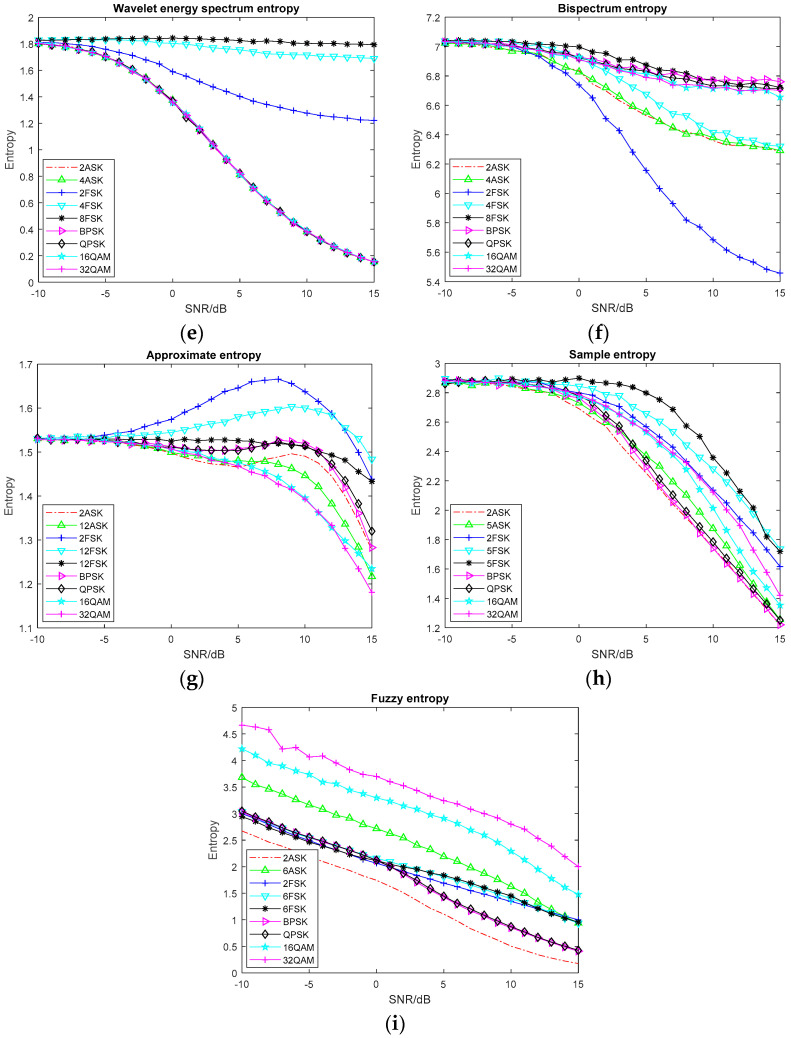
The variation curve of common information entropy with the SNR. (**a**) Power spectrum Shannon entropy; (**b**) Power spectrum exponential entropy; (**c**) Singular spectrum Shannon entropy; (**d**) Singular spectrum exponential entropy; (**e**) Wavelet energy spectrum entropy; (**f**) Bispectrum entropy; (**g**) Approximate entropy; (**h**) Sample entropy; (**i**) Fuzzy entropy.

**Figure 3:**
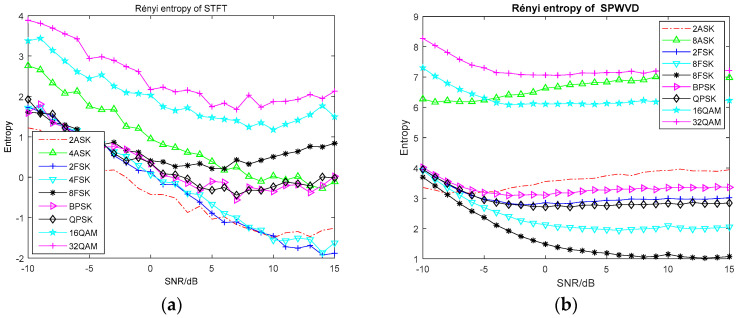

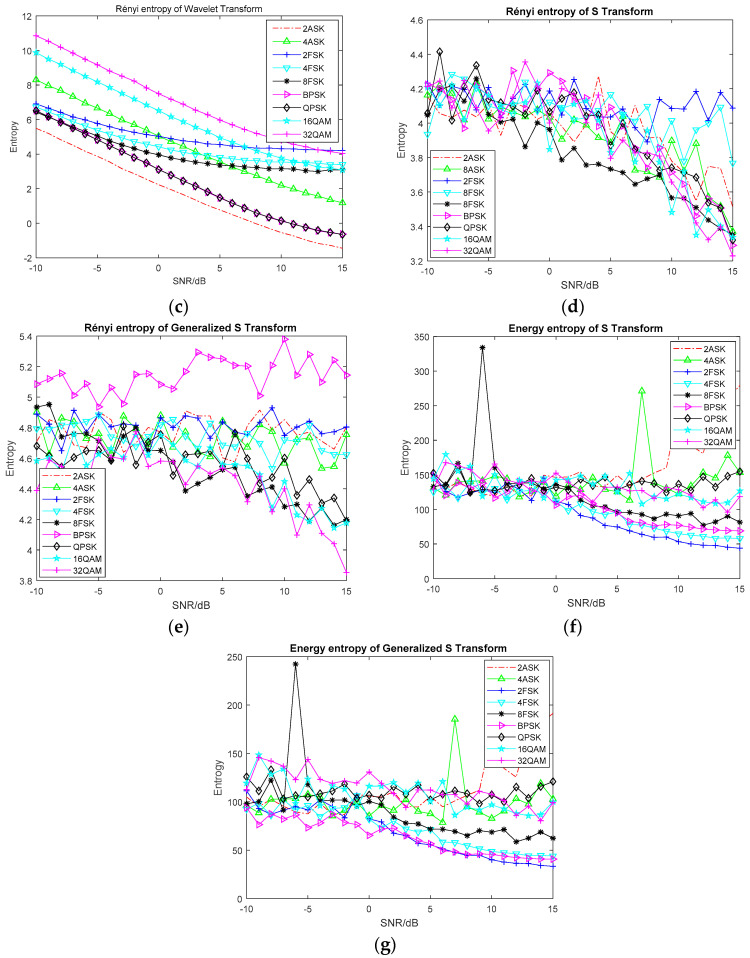
The variation curve of information entropy based on time-frequency analysis with the SNR. (**a**) Rényi entropy of STFT; (**b**) Rényi entropy of SPWVD; (**c**) Rényi entropy of Wavelet Transform; (**d**) Rényi entropy of S Transform; (**e**) Rényi entropy of Generalized S Transform; (**f**) Energy entropy of S Transform; (**g**) Energy entropy of Generalized S Transform.

For the entropy feature extraction, Monte Carlo experiments are performed 100 times on each signal at different SNRs, and the mean value of its information entropy is obtained. The variation curve of common information entropy with the SNR is shown in Figure 2. The variation curve of information entropy based on time-frequency analysis with the SNR is shown in Figure 3. The complexity of the 16 entropy features is evaluated by running each entropy feature once. The simulation time of different entropy features is shown in Table 1.

From Figure 2, we can see that most of the entropy decreases with the increase in SNR, and finally begins to stabilize. This is because as the SNR increases, the degree of signal disturbance decreases and when the SNR of the signal reaches a certain level, the change of entropy value is mainly caused by the randomness of signal symbols. As shown in Figure 2a,b, the power spectrum Shannon entropy has good discrimination on 2ASK, 4ASK, 2FSK and 8FSK signals and it easily classifies these signals. The power spectrum exponential entropy has good discrimination on 2ASK, 4ASK and 2FSK signals, the distance between other signals is relatively small, and the entropy value does not change significantly with the SNR of the signal. Compared with the power spectrum Shannon entropy, the power spectrum exponential entropy has poor classification ability for different modulation signals. Figure 2c,d shows that the singular spectrum Shannon entropy has good discrimination on MASK, MFSK and BPSK signals. However, the aliasing between QPSK and QAM signals is more serious and it is difficult to separate them by singular spectrum Shannon entropy. Compared with the singular spectrum Shannon entropy, the singular spectrum exponential entropy does not significantly improve the differentiation ability as it still cannot effectively distinguish the QPSK and QAM signals.

Figure 2e shows that the wavelet energy spectrum entropy has good discrimination on MFSK signals, and the distance between the signals is large, but it has poor ability to distinguish other signals. Figure 2f demonstrates that the distance between the bispectrum entropy values of digital signals is small, and the bispectrum entropy curve is crossed, which reveals that the bispectrum entropy feature is not effective. Also, the fluctuation of the entropy curve is large, that is, the stability of the bispectrum entropy feature is not good. In Figure 2g, the approximate entropy feature of different signals shows serious aliasing. With the increase in SNR, the distinction between entropy features of each signal is improved, but it is still dense. The approximate entropy curve is crossed, which shows that the approximate entropy feature is not effective. In Figure 2h, there is a certain degree of cross phenomenon in the sample entropy curve, and the distance of the sample entropy of digital signals is small, indicating that the sample entropy feature is not effective. Figure 2i shows that compared to the problem that the sample entropy has a small class spacing of various digital signals, fuzzy entropy is able to overcome the deficiencies of sample entropy and has good discrimination on 2ASK, 16QAM and 32QAM signals. However, for other digital signals, there is still a crossover and the problem of low differentiation, so the effect is not good.

From Figure 3a, we can see that the Rényi entropy of STFT has good discrimination on 16QAM and 32QAM signals and can realize the classification of QAM signals. However, the effect on other signals is not satisfactory, and there is a certain degree of crossover between each signal, which shows it cannot distinguish the signals effectively. Figure 3b shows the Rényi entropy of SPWVD has stable features and small fluctuations, and it has good discrimination on 16QAM, 32QAM, 4ASK, 4FSK and 8FSK signals, while it is slightly weaker for other signals. As seen in Figure 3c, the Rényi entropy of Wavelet Transform enters a stable state of change from a low SNR, and the distance between signals is also large, which makes it easy to distinguish signals. However, due to the serious crossover problem between MFSK signals and other signals, the efficiency of Rényi entropy of Wavelet Transform is reduced. From Figure 3d, for the Rényi entropy of S Transform, the aliasing between signals is more serious, the existence of multiple cross terms makes the signal extraction worse, and the fluctuation of entropy value is large. In Figure 3e, the Rényi entropy of Generalized S Transform shows a good effect on the separation of BPSK, but there are also crossover problems for other signals. The energy entropy shown in Figure 3f,g, effectively discriminates 2FSK, 4FAK, 8FSK and BPSK signals at high SNR, but the crossover of other signals is more serious. At the same time, the aliasing is serious at low SNR, which makes it difficult to distinguish the signals. The entropy values are generated by the same set of data, so there are the sudden high picks at the same SNR. Compared with Figure 3d,e, there are no picks in the Rényi entropy of S Transform and Generalized S Transform. Therefore, we think the reason for the sudden high picks is mainly due to the calculation of energy entropy. When we calculate the energy entropy, we divide the time-frequency matrix into uniform sub-matrices first, and then calculate the energy of each sub-matrix. Finally, the energy entropy is obtained by the ratio of the energy of each sub-matrix to the total energy. We think the process of sub-matrix division and the size of sub-matrix affect the existence of picks. High picks occur when the difference in the energy between the sub-matrices is too large.

Table 1 shows that the simulation time of different entropy features varies greatly. Among them, the power spectral entropy, singular spectral entropy, wavelet energy spectrum entropy, bispectrum entropy, Rényi entropy of S Transform, Rényi entropy of Generalized S Transform and energy entropy run faster and have low complexity. Approximate entropy, Sample entropy, fuzzy entropy, Rényi entropy of STFT, Rényi entropy of SPWVD, Rényi entropy of Wavelet Transform run at a slower speed, and the approximate entropy runs at the slowest speed, which runs more than 3400 times slower than the power spectrum Shannon entropy which has the fastest simulation speed. So, when the effect of approximate entropy feature extraction is not ideal, we can consider abandoning the feature to improve the simulation speed of feature extraction.

**Figure 4:**
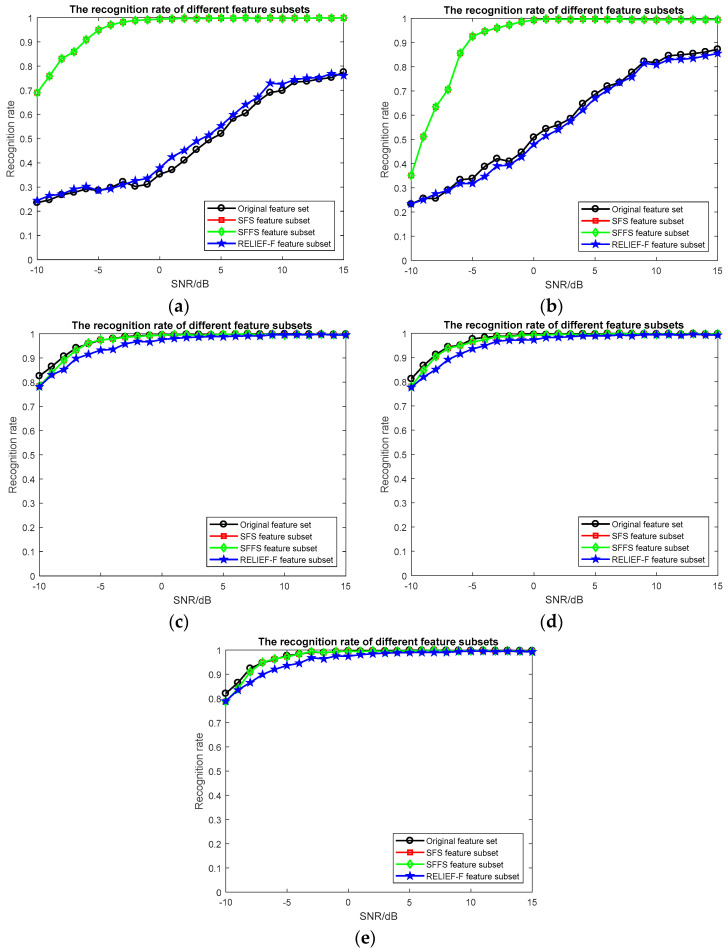
The recognition rate of feature subsets obtained by different feature selection algorithm at different SNRs. (**a**) KNN classifier; (**b**) SVM classifier; (**c**) Adaboost classifier; (**d**) GBDT classifier; (**e**) XGBoost classifier.

**Figure 5:**
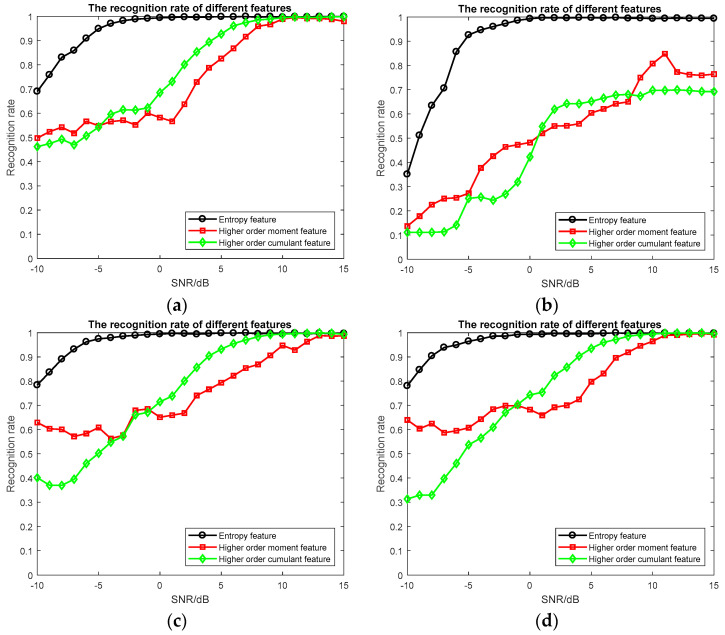

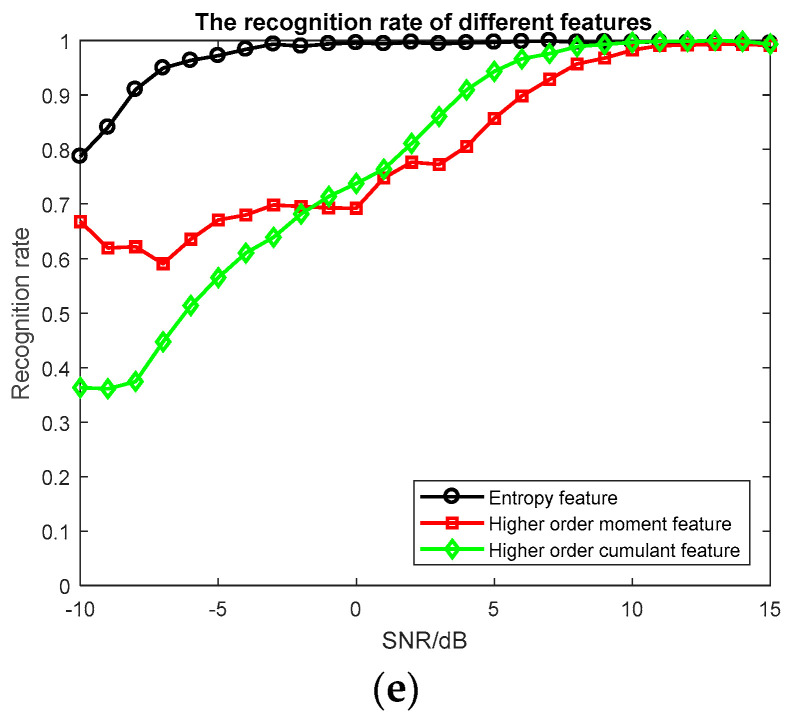
The recognition rate of different features at different SNRs. (**a**) KNN classifier; (**b**) SVM classifier; (**c**) Adaboost classifier; (**d**) GBDT classifier; (**e**) XGBoost classifier.

For the feature selection algorithm, we evaluated three aspects: the size of the feature subset, the accuracy of the classifier and the real-time performance of the algorithm. (1) The size of feature subset: the size of feature subsets obtained by different feature selection algorithms is shown in Table 2. (2) The accuracy of the classifier: It is generally considered that the accuracy of the classifier is the most important indicator for evaluating a feature selection algorithm. The recognition rate of feature subsets obtained by different feature selection algorithm is shown in Table 3. The recognition rate of feature subsets obtained by different feature selection algorithms at different SNRs is shown is Figure 4. (3) The real-time performance of the algorithm: the simulation time of different feature selection algorithms is shown in Table 4. The simulation time of different classifiers of different feature selection algorithms is shown in Table 5. To compare the entropy features, we experimented with the higher order moment features and the higher order cumulant features. The recognition rate of different features at different SNRs is shown is Figure 5.

The feature subset of each feature selection algorithm is as follows:

SFS algorithm selected 7 features: Rényi entropy of SPWVD, power spectrum Shannon entropy, wavelet energy spectrum entropy, singular spectrum Shannon entropy, singular spectrum exponential entropy, approximate entropy, power spectrum exponential entropy.

SFFS algorithm selected 7 features: Rényi entropy of SPWVD, power spectrum Shannon entropy, wavelet energy spectrum entropy, singular spectrum Shannon entropy, singular spectrum exponential entropy, approximate entropy, power spectrum exponential entropy.

RELIEF-F algorithm selected 6 features: Rényi entropy of Wavelet Transform, wavelet energy spectrum entropy, Rényi entropy of SPWVD, power spectrum Shannon entropy, energy entropy of S Transform, energy entropy of Generalized S Transform.

From Table 3, we can see that for the original feature set, the recognition rate of the traditional classifier KNN and SVM is not high, which means that 16 extracted entropy features are not suitable for the classification of KNN and SVM classifiers, and there is redundancy between the entropy features. The recognition rate of Adaboost, GBDT and XGBoost classifiers is higher, because the classifier itself has strong learning ability. For the feature subset of the SFS and SFFS algorithms, the recognition rate of the traditional classifiers, KNN and SVM has significantly improved, with an increase of 48% for KNN and 34% for SVM, which shows that the SFS and SFFS algorithms can extract more valuable features for classification. The recognition rate of Adaboost, GBDT and XGBoost classifiers is slightly lower, with a decrease of 0.44% for Adaboost, 0.43% for GBDT and 0.34% for XGBoost. Compared with the recognition rate of the original 16 features, seven features selected by SFS and SFFS algorithms can achieve similar recognition results, can reduce the computational complexity of the classifier and improve the running speed. Therefore, the SFS and SFFS algorithms have good feature selection effects. For the feature subset of the RELIEF-F algorithm, the recognition rate of each classifier is similar to the recognition rate of the original feature set, but the effect of the KNN classifier is improved, although the improvement is very small, thus the algorithm is not as good as the SFS and SFFS algorithms.

From Figure 4a, we can see that for KNN classifier, the recognition rate of feature subset of SFS, SFFS, RELIEF-F algorithms is higher than that of the original feature set. At −10 dB, the recognition rate of the original feature set is 23%, the recognition rate of RELIEF-F algorithm is 24%, which increased by 1%, while the recognition rate of the SFS and SFFS algorithm is 69%, an increase of 46%. At 15 dB, the recognition rate of the original feature set is 78%, the recognition rate of RELIEF-F algorithm is 76%, a decrease of 2%, and the recognition rate of the SFS and SFFS algorithms is 100%, an increase of 22%.

From Figure 4b, we can see that for the SVM classifier, the recognition rate of the feature subset of the SFS and SFFS algorithms is higher than the recognition rate of the original feature set, and the recognition rate of the feature subset of the RELIEF-F algorithm is lower than the recognition rate of the original feature set. At −10 dB, the recognition rate of the original feature set is 23%, the recognition rate of the RELIEF-F algorithm is 23%, and the result is similar, while the recognition rate of SFS and SFFS algorithms is 35%, which is an increase of 12%. At 15 dB, the recognition rate of the original feature set is 87%, the recognition rate of the RELIEF-F algorithm is 86%, which is a decrease of 1%, while the recognition rate of the SFS and SFFS algorithms is 99%, which is an increase of 12%. Compared with the KNN classifier, the SFS and SFFS algorithms have poor classification results at low SNRs and the RELIEF-F algorithm performs better at high SNRs. 

Figure 4c shows that for the Adaboost classifier, the recognition rate of feature subset of SFS and SFFS algorithms is lower than the recognition rate of the original feature set at low SNRs, and it has the same recognition rate with the original feature set at −6 dB, the recognition rate of the feature subset of the RELIEF-F algorithm is lower than the recognition rate of the original feature set and the feature subset of SFS and SFFS algorithms. At −10 dB, the recognition rate of the original feature set is 82%, and the recognition rate of the SFS, SFFS and RELIEF-F algorithms is 78%, which is a decrease of 4%.

In Figure 4d, we can see that for the GBDT classifier, the recognition rate of the feature subset of the SFS and SFFS algorithms is lower than the recognition rate of the original feature set at low SNRs, and it has the same recognition rate as the original feature set at 1 dB. The recognition rate of the feature subset of the RELIEF-F algorithm is lower than the recognition rate of the original feature set and the feature subset of the SFS and SFFS algorithms. At −10 dB, the recognition rate of the original feature set is 81%, and the recognition rate of the SFS, SFFS and RELIEF-F algorithms is 78%, which decreased by 3%.

From Figure 4e, we can see that for the XGBoost classifier, the recognition rate of the feature subset of the SFS and SFFS algorithms is lower than the recognition rate of the original feature set at low SNRs, and the recognition rate is the same as the original feature set at −4 dB. The recognition rate of the feature subset of the RELIEF-F algorithm is lower than the recognition rate of the original feature set and the feature subset of SFS and SFFS algorithms. At −10 dB, the recognition rate of the original feature set is 82%, and the recognition rate of the SFS, SFFS and RELIEF-F algorithms is 79%, which decreased by 3%.

Table 4 shows that the RELIEF-F algorithm has the shortest simulation time and the SFFS algorithm has the longest simulation time. Among the three feature selection algorithms, the RELIEF-F algorithm belongs to the Filter method, which has the highest operational efficiency and the shortest time required. This is the advantage of the Filter method. However, the feature subset obtained by the RELIEF-F algorithm is obviously lower in classification accuracy than the SFS and SFFS algorithms. The SFS and SFFS algorithms belong to the Wrapper method, which is a nested classifier, and it has relatively low operational efficiency and the longest time required However, the accuracy is higher than that of the RELIEF-F algorithm.

From Table 5, we can see that the simulation time classified by the feature subset selected by feature selection algorithms is, smaller in most cases than the simulation time classified by the original feature set, indicating that feature selection can reduce the computational complexity of the classifier and increase the running speed. The feature subset of the SFS and SFFS algorithms can save half the runtime of the original feature set. The RELIEF-F algorithm has a shorter runtime than the SFS and SFFS algorithms, but has the longest simulation time in SVM. The reason is that the distribution of features is chaotic and it is difficult to construct the hyperplane. Therefore, the feature subset of SFS and SFFS algorithms is the best.

From Figure 5, we can see that for each classifier, the recognition rate of entropy features is higher than the recognition rate of higher order moment features and higher order cumulant features. At low SNR, the recognition rate of higher order moment features is greater than the recognition rate of higher order cumulant features. At high SNR, the recognition rate of higher order cumulant features is higher than the recognition rate of higher order moment features. However, for SVM the recognition rate of higher-order moment features is higher at high SNR. 

## 4. Conclusions

This paper mainly studies the modulation signal recognition method based on information entropy and ensemble learning. First of all, according to the mathematical model of information entropy, this paper realizes the simulation of sixteen kinds of information entropy features of nine kinds of digital modulation signals. The selected information entropy is rich in types and contains Rényi entropy and energy entropy based on S Transform and Generalized S Transform. Because of the wide variety of available information entropy and the difficulty of determining the types of information entropy for the classification of nine kinds of digital modulation signals by the simulation results of entropy variation, three feature selection algorithms were proposed to select the optimal information entropy feature subset. We verified the effectiveness of the algorithm through the simulation of these three feature selection algorithms: the SFS algorithm, SFFS algorithm and RELIEF-F algorithm. Five classifiers including the KNN classifier, SVM classifier, Adaboost classifier, GBDT classifier and XGBoost classifier were used to classify the original feature set and feature subsets of the SFS algorithm, SFFS algorithm and RELIEF-F algorithm. 

The simulation results show that for the feature subset of the SFS and SFFS algorithm, the recognition rate of traditional classifier KNN and SVM significantly improved, with an increase of 48% for KNN and 34% for SVM, which shows that the SFS and SFFS algorithms can extract more valuable features for classification. The recognition rate of Adaboost, GBDT and XGBoost classifiers is slightly lower, with a decrease of 0.44% for Adaboost, 0.43% for GBDT and 0.34% for XGBoost. Compared with the recognition rate of the original 16 features, seven features selected by SFS and SFFS algorithms achieved similar recognition results, and reduced the computational complexity of the classifier and improved the running speed. Therefore, the SFS and SFFS algorithms have good feature selection effect. The results show that the simulation time classified by the feature subset selected by the feature selection algorithm, in most cases is smaller than the simulation time classified by the original feature set This indicates that the feature selection can reduce the computational complexity of the classifier and increase the running speed. The feature subset of SFS and SFFS algorithms can save half the runtime of the original feature set. Combined with the simulation time and recognition rate, SFS and SFFS algorithms have the best selection effect. The best overall recognition rate of the XGBoost classifier can reach 97.74% and more than 82% at −10 dB.

However, the algorithm put forward in this paper still has limitations. The SFFS algorithm includes or excludes a feature every time and has no floating value, easily falls into the local optimal solution, and as the number of features increases the complexity of the algorithm significantly increases. How to achieve the selection of the floating value of the features included or excluded and how to reduce the number of searches are issues worth studying in the future.

## Figures and Tables

**Table 1:** The simulation time of different entropy features (s).

Entropy	Time
Power spectrum Shannon entropy	0.199
Power spectrum exponential entropy	0.210
Singular spectrum Shannon entropy	0.205
Singular spectrum exponential entropy	0.204
Wavelet energy spectrum entropy	0.558
Bispectrum entropy	2.414
Approximate entropy	683.003
Sample entropy	396.102
Fuzzy entropy	428.461
Rényi entropy of STFT	162.988
Rényi entropy of SPWVD	156.508
Rényi entropy of Wavelet Transform	166.227
Rényi entropy of S Transform	10.224
Rényi entropy of Generalized S Transform	9.986
Energy entropy of S Transform	7.043
Energy entropy of Generalized S Transform	6.974

**Table 2:** The size of feature subsets obtained by different feature selection algorithms.

Algorithm	No	SFS	SFFS	RELIEF-F
Features	16	7	7	6

**Table 3:** The recognition rate of feature subsets obtained by different feature selection algorithm.

Algorithm	NO	SFS/SFFS	RELIEF-F
KNN	47.76%	**95.71%**	49.53%
SVM	57.93%	**91.48%**	56.39%
Adaboost	**97.63%**	97.19%	95.70%
GBDT	**97.59%**	97.16%	95.70%
XGBoost	**97.74%**	97.40%	95.91%

**Table 4:** The simulation time of different feature selection algorithms (s).

Algorithm	Time
SFS	465.909
SFFS	735.793
RELIEF-F	3.467

**Table 5:** The simulation time of different classifiers of different feature selection algorithms (s).

Algorithm	NO	SFS/SFFS	RELIEF-F
KNN	5.179	2.075	1.856
SVM	2352.019	124.972	2495.665
Adaboost	11.544	5.507	4.914
GBDT	36.644	18.049	17.659
XGBoost	13.276	7.784	6.973
